# Research on advanced intervention using novel bone marrOW stem cell (RAINBOW): a study protocol for a phase I, open-label, uncontrolled, dose-response trial of autologous bone marrow stromal cell transplantation in patients with acute ischemic stroke

**DOI:** 10.1186/s12883-017-0955-6

**Published:** 2017-09-08

**Authors:** Hideo Shichinohe, Masahito Kawabori, Hiroaki Iijima, Tuyoshi Teramoto, Takeo Abumiya, Naoki Nakayama, Ken Kazumata, Shunsuke Terasaka, Teruyo Arato, Kiyohiro Houkin

**Affiliations:** 1 Department of Neurosurgery, Hokkaido University Graduate School of Medicine, Sapporo, Japan; 2 Clinical Research and Medical Innovation Center, Hokkaido University Hospital, N14 W5, Kita-ku, Sapporo, 060-8648 Japan

**Keywords:** Acute ischemic stroke, Bone marrow stromal cells, Cell therapy, Regenerative medicine, Intraparenchymal injection, Platelet lysate, Bio-imaging

## Abstract

**Background:**

Stroke is a leading cause of death and disability, and despite intensive research, few treatment options exist. However, a recent breakthrough in cell therapy is expected to reverse the neurological sequelae of stroke. Although some pioneer studies on the use of cell therapy for treating stroke have been reported, certain problems remain unsolved. Recent studies have demonstrated that bone marrow stromal cells (BMSCs) have therapeutic potential against stroke. We investigated the use of autologous BMSC transplantation as a next-generation cell therapy for treating stroke. In this article, we introduce the protocol of a new clinical trial, the Research on Advanced Intervention using Novel Bone marrOW stem cell (RAINBOW).

**Methods/design:**

RAINBOW is a phase 1, open-label, uncontrolled, dose-response study, with the primary aim to determine the safety of the autologous BMSC product HUNS001–01 when administered to patients with acute ischemic stroke. Estimated enrollment is 6–10 patients suffering from moderate to severe neurological deficits. Approximately 50 mL of the bone marrow is extracted from the iliac bone of each patient 15 days or later from the onset. BMSCs are cultured with allogeneic human platelet lysate (PL) as a substitute for fetal calf serum and are labeled with superparamagnetic iron oxide for cell tracking using magnetic resonance imaging (MRI). HUNS001–01 is stereotactically administered around the area of infarction in the subacute phase. Each patient will be administered a dose of 20 or 50 million cells. Neurological scoring, MRI for cell tracking, ^18^F–fuorodeoxyglucose positron emission tomography, and ^123^I–Iomazenil single­photon emission computed tomography will be performed for 1 year after the administration.

**Discussion:**

This is a first-in-human trial for HUNS001–01 to the patients with acute ischemic stroke. We expect that intraparenchymal injection can be a more favorable method for cell delivery to the lesion and improvement of the motor function than intravenous infusion. Moreover, it is expected that the bio-imaging techniques can clarify the therapeutic mechanisms.

**Trial registration:**

The trial was registered at The University Hospital Medical Information Network on February 22, 2017 (UNIN ID: UMIN000026130). The findings of this trial will be disseminated to patients and through peer-reviewed publications and international presentations.

## Background

Since Azizi et al. published the first report on bone marrow stromal cell (BMSC) transplantation in 1998 [1], BMSCs have been considered as a promising cell source for central nervous system (CNS) regeneration. Some well-known advantages of BMSCs are the ease of their harvest, availability of autologous cells, absence of immunological rejection or tumorigenesis, and freedom from ethical problems [2]. Several articles have reported that BMSCs can survive in the host CNS, migrate to the lesion, and elicit neuroprotective effects when transplanted into animal models of CNS disease [3].

BMSCs originate from bone marrow mononuclear cells (BMMNCs). The floating cells are removed from BMMNCs, and only cells adhering to the floor of the flask are cultured for several weeks to obtain BMSCs [4]. A subpopulation of BMSCs, also termed mesenchymal stem cells, can differentiate into bone, cartilage, and fat. Moreover, there are many reports on their potential to differentiate into other lineages, including neural cells, via so-called transdifferentiation. Many researchers have reported how BMSCs could protect injured CNS. At first, their transdifferentiation to neuronal cells, endothelial cells, or pericyte was noticed. On the other hand, it has been known that they can promote neurogenesis, axonal elongation, vasculogenesis, and so on, due to their secretion of some growth factors or cytokines, so-called nursing effect [5, 6].

The first clinical trial using autologous BMSC therapy in patients with stroke was reported in 2005. Bang et al. showed the feasibility and safety of the therapy [7]. They adopted cell culture methods with fetal calf serum (FCS) and intravenous administration in the protocol. Regrettably, they reported no significant neurological recovery using the Barthel Index and modified Rankin Scale. We hypothesized that some unsolved problems regarding clinical application remained, including safety of the cell culture, suitable delivery routes, cell tracking after the transplantation, and monitoring the effects of intervention [8]. We aimed to solve these problems to develop the next generation of BMSC therapy for stroke. We hypothesize that xeno-free cell culture, direct cell administration near the lesion, and bioimaging for the therapeutic effects are indispensable for the next generation of BMSC therapy [8].

We have reported translational research on human BMSC transplantation in animal stroke models. Human BMSCs were cultured with human platelet lysate (hPL) instead of FCS [9, 10]. The cells were injected stereotactically into rat ischemic brains [9, 10]. In advance, the donor cells were labeled with super­paramagnetic iron oxide (SPIO) for cell tracking using magnetic resonance imaging (MRI) [10]. After the transplantation, ^18^F- fluorodeoxyglucose positron emission tomography (FDG-PET) and ^123^I–Iomazenil single­photon emission computed tomography (IMZ­SPECT) were performed to analyze cellular function and metabolism in the host brain [11, 12]. There were no differences in the surface markers and cell proliferation between cells culture in hPL and FCS [9]. Although a rotarod test showed that motor function deteriorated in rats suffering from permanent middle cerebral artery occlusion, a BMSC-hPL transplantation enhanced recovery of the motor function significantly [9]. MRI demonstrated that the SPIO-BMSCs aggressively migrated towards the lesion [10]. Moreover, FDG-PET and IMZ-SPECT showed that BMSC transplantation promoted recovery of the glucose utilization and the binding potential of iomazenil, respectively, in the peri-infarct area [11, 12]. Histological analysis supported the findings on MRI and showed an inclination for neural differentiation of donor cells [10, 13, 14].

We used these results to determine the optimal study design of our new clinical trial, Research on Advanced Intervention using Novel Bone marrOW stem cell (RAINBOW). The trial is a Phase I study to assess the potential benefits of autologous BMSC product (code name: HUNS001–01) administration to patients with acute ischemic stroke. We aim to evaluate its safety, feasibility, and efficacy. Autologous BMSC were cultured with allogeneic PL in the cell processing center (CPC) according to good manufacturing practice (GMP). The cells were labeled with SPIO. They were injected around the infarct stereotactically. After administration, we will perform MRI for cell tracking and FDG-PET and IMZ-SPECT for the analysis of the therapeutic effect. In addition, we hope that this study proves helpful in clarifying the therapeutic mechanisms. In the study, we expect that the findings of SPIO-labeling/MRI will help us to choose the suitable transplant location. About the therapeutic mechanisms, for instance, if MRI showed that the cluster of donor cells moved to SVZ, it might be an evidence for stimulation of endogenous neuronal regeneration. And FDG-PET and IMZ-SPECT may show the relation between the transplant location and the reaction of the host brain, for examples, brain metabolism may be increased in the contralateral hemisphere to the transplant location.

## Methods/design

### Study design

**Fig. 1:**
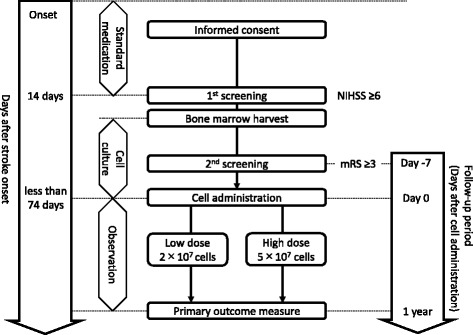
The detailed trial flow of RAINBOW

This study is an open-label, uncontrolled, dose-response, single-center, phase I clinical trial. The subjects will receive standard medication for 14 days after the onset of their stroke. The patients are then screened, and bone marrow harvest is performed as soon as possible. The BMSCs are cultured with allogeneic PL, and HUNS001–01 is manufactured from autologous BMSCs in the CPC. Once the preparation of HUNS001–01 is almost complete, the administration date is planned, and a second screening is performed 7 days before the administration. If the subject meets the requirements, HUNS001–01 (cell dose: 2 × 10^7^ or 5 × 10^7^ cells) is injected into the brain stereotactically. Follow-up is done for 1 year after the administration. The detailed trial flow is described in Fig. 1. This study is conducted in the Department of Neurosurgery, Hokkaido University Hospital, Sapporo, Japan. The trial was registered at The University Hospital Medical Information Network on February 22, 2017 (UMIN ID: UMIN000026130). The total study period is approximately 3 years, between March 2017 and August 2020.

### Study population

Inclusion criteria (First screening; 14 days after the onset of stroke).

- Male or female subjects between 20 and 79 years old
- Informed consent within 14 days after the onset of stroke
- Clinical diagnosis of cerebral ischemic stroke in the internal carotid arterial region
- Subjects with a modified Rankin Scale (mRS) of 0 or 1 before the onset of stroke
- Subjects who can give informed consent; if insufficiently able, a legal representative is needed
- Subjects with moderate or severe neurological symptoms; National Institute of Health Stroke Scale (NIHSS) ≥ 6, (however, the partial score in both “Motor Arm” and “Motor Leg” must be ≥6)

(Second screening; 7 days before the administration of HUNS001–01).

- HUNS001–01 must be available within 74 days after the onset
- Subjects with moderate or severe neurological deficit; mRS ≥ 3

Exclusion criteria (First screening; 14 days after the onset of stroke)

- Occurrence of severe hemorrhagic transformation of ischemic stroke
- Subjects in a coma or a deep coma with a JCS-200 or JCS-300 score evaluated by the Japan Coma Scale
- Severe anemia (hemoglobin < 10.0 g/dL) or thrombocytopenia (platelet count < 100,000/mm^3^)
- Severe heart disease (e.g., ischemic heart disease, heart failure)
- Significant abnormalities in laboratory tests:
  a. >3 × upper limit of normal (ULN) for alanine aminotransferase or aspartate aminotransferase.
  b. >1.5 × ULN for total bilirubin.
  c. >1.5 × ULN for serum creatinine
- Uncontrolled hypertension, despite antihypertensive therapy
- History of malignancy of any type
- Carriers of any of the following infectious diseases: Syphilis, hepatitis B virus (HBV), hepatitis C virus (HCV), human immunodeficiency virus (HIV)-1/2, human T cell leukemia virus (HTLV)-1, or parvovirus B19
- Subjects who are pregnant or want to have children
- History of seizure or administration of any antiepileptic drugs
- Contraindication for fosphenytoin sodium hydrate (Fostoin®, Eisai Co., Ltd., Tokyo, Japan)
- Serious allergy to any possible residues in the test product (e.g., any biomaterials used in manufacturing process, gentamicin sulfate, ferucarbotran) or any agents used for the administration of the test product or for inspections during the trial
- Contraindication for MRI (e.g., a pacemaker, metallic artificial heart valves, an implantable cardioverter defibrillator)
- Subjects who are inappropriate for this trial based on the judgment of the primary investigator or other investigators; for example, if other intracranial disorders were diagnosed due to history, symptoms, or neuroradiological findings during the trial, the primary investigator or other investigators will make a decision after reviewing the risks and benefits.

(Second screening; 7 days before the administration)

- Occurrence of severe hemorrhagic transformation of ischemic stroke
- Subjects in a coma or a deep coma with a JCS-200 or JCS-300 score evaluated by Japan Coma Scale
- Severe anemia (hemoglobin < 10.0 g/dL) or thrombocytopenia (platelet count < 100,000/mm^3^)
- Severe heart disease (e.g., ischemic heart disease, heart failure)
- Significant abnormal laboratory tests:
  a. >3 × ULN for alanine aminotransferase or aspartate aminotransferase.
  b. >1.5 × ULN for total bilirubin.
  c. >1.5 × ULN for serum creatinine
- Uncontrolled hypertension, despite antihypertensive therapy
- Subjects who are inappropriate for this trial based on the judgment of the primary investigator or other investigators; for example, if other intracranial disorders were diagnosed due to history, symptoms, or neuroradiological findings during the trial, the primary investigator or other investigators will make a decision after reviewing the risks and benefits.

### Sample size and statistical analysis

Since this trial is a phase I pilot study, each group is composed of three subjects to examine the safety of HUNS001–01 administration. The sample size of the trial was based on the traditional 3 + 3 dose escalation design. The first three subjects are included in the low-dose group (cell dose: 2 × 10^7^ cells), and the following three patients are included in the high-dose group (cell dose: 5 × 10^7^ cells). However, if the cultured BMSCs are inadequate for the high-dose group on 74 days after the onset of stroke, a subject could be moved to the low-dose group, if possible. In that case, the number of subjects will exceed six, and the last subject is the third subject in the high-dose group. The total number of subjects is limited to 10 if a third subject in the high-dose group is not accomplished.

Continuous data will be presented as means and standard deviations. Categorical data will be presented as absolute frequencies or relative percentages.

### Characteristics of HUNS001–01

HUNS001–01 consists of plastic-adherent fibroblast-like cells. The phenotype of these cells is positivity for CD44, CD90, CD105, CD106, CD146, and CD 166, and negativity for CD19, CD34, and CD45. The expression of markers are consistent with the mesenchymal stromal cell phenotype according to the position statement of the International Society for Cellular Therapy (ISCT) [15].

### Preparation of HUNS001–01

The cell source for HUNS001–01 is obtained by extracting approximately 50 mL of bone marrow from each subject. The bone marrow is brought to the CPC at Hokkaido University Hospital, and the following processes are performed in a closed operation system (CPWS System Cell Processing Work Station, Panasonic Healthcare Co., Tokyo, Japan). BMMNCs are isolated via density-gradient centrifugation with Ficoll-Hypaque® (Pharmacia, Uppsala, Sweden), and 1 × 10^7^ cells are plated in a 175 cm^2^ uncoated flask (Easy Flask 159,910; Nunc) with 25 mL of αMEM with 10% hPL derived from healthy volunteers and 40 μg/mL of gentamicin sulfate. About the preparation of hPL, our previous report about safety and efficiency of hPL should be referred to [4]. After 24 h, nonadherent cells are removed by changing the medium. The culture medium is replaced 2 times a week. The BMSCs are passed two or three times for the subsequent procedure. In order to label the cells for MRI tracking, 1 μL/mL ferucarbotran (27.9 μg Fe/mL, Resovist®, Fuji lm RI Pharma Co., Ltd., Tokyo, Japan), a SPIO agent, is added into the culture medium and incubated with the BMSCs for 24 h before the cell injection procedure. The SPIO-labeled BMSCs in flasks are lifted using TrypLe Select® (a recombinant trypsin substitute, Gibco) and centrifuged. The supernatant is decanted and the cells are gently resuspended in Artcereb® (irrigation and perfusion solution used for cerebrospinal surgery; Otsuka Pharmaceutical Factory, Inc., Naruto, Japan) to a concentration of 5 × 10^7^ cells/mL.

### Intervention

On the day when HUNS001–01 is manufactured, the cell product will be administered to the subject. A dose of 2 × 10^7^ cells (400 μL) will be administered in the low-dose group and a dose of 5 × 10^7^ cells (1000 μL) will be administered in the high-dose group. HUNS001–01 is implanted using MRI stereotactic technique to define the target sites in the normal white matter around the lesion. The number of target sites is one or two in the low-dose group, and two or three in the high-dose group. The subject has a one or two burr-hole craniotomy under local anesthesia and sedation. Using 2.1-mm outer diameter stereotactic cannula, 200 to 500 μL of the product is injected over a period of 5 min at each site. The cannula is removed after 5 min after finishing the injection at each site. In the perioperative period, all subjects take fosphenytoin sodium hydrate to prevent epileptic seizure due to the procedure.

### End point of the study

#### Primary end point

- Safety of HUNS001–01 administration for 1 year after the intervention: frequency of Adverse Event (AE)

#### Secondary end points

- Safety of HUNS001–01 administration for 30 days after the intervention: frequency of Adverse Event (AE)
- Safety of bone marrow aspiration: frequency of Adverse Event (AE)
- Frequency of the defects of HUNS001–01 at manufacturing
- Improvement of stroke symptoms for 1 year after the intervention using the following assessment scales:
  - ational Institute of Health Stroke Scale (NIHSS)
  - modified Rankin Scale (mRS)
  - Functional Independence Measure (FIM)
  - Barthel Index
  - Fugl-Meyer Assessment
- Improvement in lesion volume assessed by MRI analysis for 1 year after the intervention
- Assessment of cell distribution using MRI
- Assessment of possible functional shift for 1 year after the intervention using FDG-PET and IMZ-SPECT

### Follow-up of study

The schedule of follow-up of study is as follows (Table 1):

- Vital signs, serological, and biochemical tests: Days 0, 1, 3, 7, 14, 30, 90, 180, 360
- Urinalysis: Days 30, 90, 180, 360
- Urine hCG-β (if needed): Days 90, 180, 360
- 12-lead electrocardiogram and chest X-ray examination: Days 1, 7, 30, 90, 360
- Neurological examination (NIHSS, mRS, FIM, Barthel Index, Fugl-Meyer Assessment): Days 1, 3, 7, 14, 30, 90, 180, 360
- MRI: Days 0, 3, 7, 14, 30, 90, 180, 360
- FDG-PET and IMZ-SPECT: Days 30, 90, 360

**Table 1:** The schedule of follow-up of study

	Screening			Follow up									
	1st screening	BM hervest	2nd screening	Pre-administration	Post-administration								
	Onset to14 days		Day −7 (±1)	Day 0		Day 1	Day 3	Day 7	Day 14	Day 30 (±3)	Day 90 (±30)	Day 180 (±30)	Day 360 (±30)
Informed consent	X												
Medical history	X												
Vital signs	X	X	X	X	X	X	X	X	X	X	X	X	X
Body weight and height	X												
Serological tests	X		X		X	X	X	X	X	X	X	X	X
Biochemical tests	X		X		X	X	X	X	X	X	X	X	X
Urinalysis	X		X							X	X	X	X
Urine hCG-β (If needed)	X									X	X	X	X
Infectious disease inspection	X												
12-lead electrocardiogram	X		X			X		X		X	X		X
Chest X-ray examination	X		X			X		X		X	X		X
Neurological examination	X		X			X		X		X	X	X	X
MRI	X		X	X	X		X	X	X	X	X	X	X
FDG-PET and IMZ-SPECT			X							X	X		X
Bone marrow harvest		X											
Cell product administration				X									
Concomitant medication	X	X	X	X	X	X	X	X	X	X	X	X	X
Adverse events	X	X	X	X	X	X	X	X	X	X	X	X	X

### Data collection and monitoring

All data are collected by appointed staff members, who are approved by the Clinical Research and Medical Innovation Center, Hokkaido University Hospital. They are monitored by the Standard Operating Procedures (SOPs) of Good Clinical Practice (GCP) and the ICH guidelines.

### Ethics and dissemination

This trial is conducted following the GCP guidelines and the principles of the Declaration of Helsinki. The data obtained in this study will be disseminated in peer-reviewed journals and presented at international scientific meetings.

## Discussion

### Subject screening

Current therapeutic strategies for ischemic stroke typically aim to improve blood flow in ischemic areas or to relieve neuronal damage through neuroprotective effects before the ischemic disorder is established. However, in cell therapy, some strategies aim to resolve neurological disorders resulting from an established cerebral infarction. Autologous cell products, in particular, require time for tissue collection and manufacturing. If changes in a subject’s condition are anticipated during the manufacturing period, appropriate screening at the start of the administration, as well as at the enrollment should be established.

In our trial, NIHSS score is assessed in the first screening on day 14 after the onset. The NIHSS is used for systematic assessment of potential symptoms and signs of stroke, and is designed primarily to assess symptoms and signs in the acute phase and to determine the severity of the disease. On the other hand, mRS, which is one of the disability measures, has been widely used as an efficacy endpoint in clinical trials all over the world. In our trial, mRS is examined in the second screening, 7 days before the administration. H, the NIHSS score on admission is inadequate for the first screening. Due to the weak correlation with the mRS score in chronic phase, possible dropouts are expected at the second screening. In fact, Kimura et al. reported that approximately half of the subjects with NIHSS scores of 7–10 at admission would have an mRS score of 0–2 at discharge [16]. We adopted the NIHSS on day 14 after the onset, but not on admission, as the first screening, because it has a stronger correlation with the mRS score and other outcome scales in the chronic phase [17].

In the trial, we enrolled patients who met the inclusion criteria of a total score of NIHSS ≥6, and a “Motor Arm” and “Motor Leg” score of NIHSS ≥6 at the first screening, since the improvement of injured motor function is an anticipated result of the administration of HUNS001–01.

### Safety measures for intraparenchymal injection

Intraparenchymal injection is a technique to administer cells directly into the cerebral parenchyma. This technique can deliver numerous cells selectively to peri-infarct regions. Attention should be paid to possible complications of trepanation and cerebral puncture, such as epilepsy and hemorrhage due to cerebral injuries. In 2005, Kondziolka et al. reported a phase 2 trial with LBS-Neurons (human teratocarcinoma cell line origin, Layton BioScience, Inc.) [18]. They adopted the intraparenchymal cell transplantation technique for 14 patients with stroke. Serial evaluations demonstrated that one patient had a single seizure and one had an asymptomatic chronic subdural hematoma. In a recent report, Steinberg et al. described a phase 1/2A study, with SB623 cells (SanBio Inc., CA, USA) [19]. Eighteen patients with ischemic stroke underwent stereotactic transplantation in the chronic phase. In this trial, one patient had a single seizure and one had asymptomatic chronic subdural fluid collection.

In our protocol, all subjects will be taking fosphenytoin sodium hydrate to prevent epileptic seizures in the perioperative period. Moreover, we use a round head stereotactic cannula to prevent hemorrhagic events due to the procedure, while a thin and pointed cannula was used in the two previously mentioned trials.

The sample size of the trial was based on the traditional 3 + 3 dose escalation design. Indeed, it may be difficult to reach statistically significant differences with small number (6 to 10) of patients, but we think that such a small population can verify the safety. For examples, Savitz et al. reported the safety concerns of clinical trials about the stereotactic transplantation with LGE cells (Genvec Inc.) in only 5 stroke patients [20]. Because 2 patients had the adverse effects (the temporary worsening of motor deficits 3 weeks after transplantation and the seizures 1 week after transplantation), the trial was terminated by the FDA.

### Administration dose

In our protocol, the administration dose was determined based on our own preclinical rodent study and prior clinical trials. In our preclinical safety study, immunodeficient rats received 1 × 10^6^ BMSCs in the brain parenchyma. When the data was extrapolated, we determined that this is equivalent to an administration cell dose of 7 × 10^8^ cells in humans. Because the highest dose used in the trial is 5 × 10^7^ cells (high-dose group), which is one-fourteenth of the extrapolated dose, we concluded that this dose is adequate to investigate safety as the objects of the study.

Honmou et al. reported a phase 1/2 trial using autologous MSCs [21]. Twelve patients with acute cerebral infarction received an intravenous cell infusion (mean cell dose: 1 × 10^8^ cells). They showed improvement in neurologic symptoms in 11 patients and concluded that intravenous administration of autologous MSCs may be effective for treatment of patients with acute cerebral infarction. In our previous study on cell delivery routes, however, we observed that intraparenchymal injection is a more favorable method for cell delivery to the lesion and improvement of the motor function than intravenous infusion [22]. When only one-third dose of BMSCs (1 × 10^6^ cells) was delivered using intraparenchymal injection in a rat stroke model, the injured motor function was improved significantly compared with BMSC intravenous administration (3 × 10^6^ cells). When we extrapolated data from the preclinical findings, we determined that one-third dose of BMSC (3.3 × 10^7^ cells) would be more effective when administered via intraparenchymal injection than intravenous infusion. In our protocol, two doses of 2 × 10^7^ and 5 × 10^7^ cells were used. Thus, former is a lower dose of 3.3 × 10^7^ cells, whereas the latter is an upper dose. The significance of dose-response relationships of cell products has not been established sufficiently. The low-dose group may be more effective because of a shorter cell culture period and earlier administration. Moreover, not only cell dose, but also administration site should be optimized. In our trial, these controversial variables may be solved using bioimaging methods, cell tracking using MRI, and assessment of functional shift using FDG-PET and IMZ-SPECT.

### Time point of cell administration

In the protocol, we cannot provide the exact time point of cell administration. The autologous cells need a period for cell culture before the administration. We expect that the range of the period will be from 2 to 5 weeks, and then we will administer the cells as soon as possible. But the period may be prolonged due to some factors, for examples, aging of subjects. We set the dead line of the administration to 74 days after stroke onset, which means the culture period is set for maximum 60 days, because delayed cell administration may miss the therapeutic time window and the cells with too slow growth may be unhealthy or ineffective.

## Abbreviations

AE: Adverse Event
BMMNC: Bone marrow mononuclear cell
BMSC: Bone marrow stromal cell
CNS: Central nervous system
CPC: Cell processing center
FCS: Fetal calf serum
FDG-PET: ^18^F- fluorodeoxyglucose positron emission tomography
FIM: Functional Independence Measure
GCP: Good clinical practice
GMP: Good manufacturing practice
HBV: Hepatitis B virus
HCV: Hepatitis C virus
HIV: Human immunodeficiency virus
hPL: Human platelet lysate
HTLV: Human T cell leukemia virus
IMZ­SPECT: ^123^I–Iomazenil single­photon emission computed tomography
ISCT: International society for cellular therapy
JCS: Japan coma scale
MRI: Magnetic resonance imaging
mRS: Modified rankin scale
NIHSS: National institute of health stroke scale
RAINBOW: Research on advanced intervention using novel bone marrow stem cell
SOP: Standard Operating Procedure
SPIO: Super­paramagnetic iron oxide
ULN: Upper limit of normal
UMIN: The university hospital medical information network
